# Von Karman rotating nanofluid flow with modified Fourier law and variable characteristics in liquid and gas scenarios

**DOI:** 10.1038/s41598-021-95644-w

**Published:** 2021-08-12

**Authors:** Muhammad Ramzan, Hina Gul, M. Mursaleen, Kottakkaran Sooppy Nisar, Wasim Jamshed, Taseer Muhammad

**Affiliations:** 1 Department of Computer Science, Bahria University, Islamabad, 44000 Pakistan; 2 Department of Medical Research, China Medical University Hospital, China Medical University (Taiwan), Taichung, Taiwan; 3 Department of Mathematics, Aligarh Muslim University, Aligarh, 202002 India; 4 Department of Mathematics, College of Arts and Sciences, Prince Sattam Bin Abdulaziz University, Wadi Aldawaser, 11991 Saudi Arabia; 5 Department of Mathematics, College of Sciences, King Khalid University, Abha, 61413 Saudi Arabia

**Keywords:** Mechanical engineering, Software

## Abstract

This investigation aims to explore the temperature-dependent variable characteristics of viscosity, and thermal conductivity with modified Fourier law in a nanofluid flow over a rotating disk. The uniqueness of the envisioned mathematical model is improved with the additional impacts of the chemical reaction, non-uniform source/sink, and convective boundaries. The salient feature of the existing problem is to discuss the whole scenario with liquid and gas thermo-physical characteristics. The graphical depiction is attained for arising pertinent parameter is attained by using Bvp4c a built-in MATLAB function. The visco-thermal conduct of the gases and liquids is examined by observing the mean flow and thermal distributions for the convectively heated disk. It is followed that liquid behaves more viscous with an increase in temperature in of the gas, but an opposing tendency can be seen for the liquid. The attained results are verified when compared with a published result.

## Introduction

Heat transfer phenomenon has an important role in industrial, natural, geophysical, and bioengineering processes. Transfer of heat occurs due to temperature differences amongst two different objects or within a similar object. Fourier law (heat conduction) states that any disturbance instigated in the beginning will carry out throughout the process. To resolve this matter, Cattaneo introduced thermal relaxation time in traditional Fourier's law (heat conduction) which allows the transport of heat by waves propagating with controlled speed^1–3^. Later, Christov developed the relation proposed by Cattaneo through frame-indifferent change with upper-convected Oldroyd derivative. Such relation is entitled as Cattaneo-Christov(C–C) flux model. The theory of heat and mass fluxes for C–C in three-dimensional Oldroyd-B fluid flow past over a rotating cone is examined by Hafeez et al.^4^. Shehzad et al.^5^ studied MHD incompressible Maxwell bioconvection fluid flow past over a rotating isolated disk in the attendance of C–C heat flux. A nanofluid Buongiorno model with a gyrotactic microorganism is executed in this exploration. The numerical results are attained by engaging the Runge–Kutta-Fehlberg numerical scheme. Ramzan et al.^6^ examined the effects of melting heat transfer carbon nanotubes with C–C heat flux amidst two rotating disks. The MATLAB function bvp4c numerical procedure is adopted here. Tulu et al.^7^ studied the effects of C–C heat flux in the nanofluid flow comprising carbon nanotubes-ethylene glycol over a rotating disk. The SQL (Spectral Quasi-Linearization Method) is engaged for numerical results. Lu et al.^8^ numerically solved the mathematical model of nanofluid time-dependent flow containing both types of nanotubes between two rotating disks with C–C heat flux and the HOM-HET reaction model. The flow generated by a rotating disk with C–C heat flux is deliberated analytically by Imtiaz et al.^9^. Influence of C–C heat flux and Darcy-Forchheimer tangent hyperbolic dusty nanoliquid flow past over a stretching sheet explore by Shanaralingappa et al.^10^. Reddy et al.^11^ introduced the effects of C–C heat flux and CNT with Darcy-Forchheimer and nonlinear thermal radiation along the melting surface and slip condition. The influence of Maxwell liquid and magnetic dipole flow past over a stretching sheet is considered by Kumar et al.^12^. Lately, researchers have shown great interest in various aspects of C–C heat flux^13–16^.

Nanofluids is an emerging field of industry that has trapped the eye of many researchers who were searching for methods to make cooling procedures more efficient in the industry. Cooling of electronic devices, drug delivery systems, biological sensor systems, Automobile, Solar cell, and nuclear controlling system are the applications of nanofluid. Nanofluid is a liquid that is made by the immersion of nanoparticles with sizes less than 100 nm in fluids. A nanofluid low thermal conductivity is one of its prominent features that can restrain the performance of the transfer of heat. Due to their shortcomings in heat transfer properties, normal heat transport fluids such as H_2_O and (CH_2_OH)_2_, and oil have restricted heat transfer capabilities and are therefore unable to meet industrial cooling requirements. Ramzan et al.^17^ numerically discovered bioconvection nano liquid flow and partial slip effect due to a rotating Attia^18^ discussed the flow of the Reiner-Rivlin liquid with Ion slip and Hall current impacts past a rotating disk. An interesting result of this investigation points out that the influence of the Ion slip on the axial velocity is more prominent for Reiner-Rivlin fluid as compared to any Newtonian liquid. Abbas et al.^19^ deliberated the numerical solution of the MHD nanoliquid flow past over a rotating disk with second-order velocity slip and activation energy with entropy minimization optimization. The main finding of the envisioned model is that the entropy generation is effects by the magnetic field. The flow of Marangoni Maxwell fluid past a rotating disk accompanying the thermal radiation and activation energy is examined numerically by Devi and Mabood^20^. The salient outcome highlighting the entropy generation $(S_{G} )$ impact is that the higher estimates of the Bejan number and the fluid parameter weaken the $S_{G}$ rate. Abbasi et al.^21^ deliberated viscoelastic bio-connection nano liquid flow over a rotating convective stretching disk. The Keller Box method is used to obtain numerical results. Alumina nanomaterial with free convection in a chamber along a finned copper thermal sink studied by Sheremet et al.^22^. Impact of a chemical reaction and thermal radiation with gyrotactic microorganism and MHD nanoliquid flow examine by Bhatti et al.^23^. Comparison of heat transfer by varied shapes of copper nanoparticles flow with base liquid water is examine by Saleem et al.^24^. Ahmed et al.^25^ explored that the Unsteady heat transfer flow of MHD CNT and variable viscosity with porous media pass over a shrinking surface. Augmentation of heat transfer in micropolar hybrid nanoliquid numerically investigated by Rana et al.^26^. Analysis of nanoliquid flow of C–C heat flux for heat generation with MFD viscosity in a semi-circular cavity discussed by Dogonchi et al.^27^. Analysis of ferroliquid with constant thermal radiation and magnetic field with two circular cylinders introduced by Sadeghi et al.^28^. Examination of turbine cooling for heat transfer with a non-Newtonian liquid flow over a disk is discussed by Dogonchi and Ganji^29^. Numerical examination of $S_{G}$ in a semi-annulus permeable media of a nanofluid enclosure with different shapes of nanoparticle in the existence of a magnetic field examines by Seyyedi et al.^30^. Some current publications emphasizing various features of the nanofluids may be found in^31–38^.

The role of thermal conductivity is fundamental in the thermophysical characteristics of the fluid flow and numerous engineering processes. A good number of studies are available that focuses on constant thermal conductivity. Nevertheless, it is required to study the variable thermal conductivity. Abbasi and Shehzad^39^ examine the impacts of heat transfer analysis with variable thermal conductivity and C–C heat flux. It is detected that the thermal profile is more dominant in the case of the Fourier law of heat conduction as compared to C–C heat flux. Maleque and Sattar^40^ examined the variable properties and Hall current on a laminar convective liquid flow by a rotating disk in a porous media. Naganthran et al.^41^ introduced dual effects liquid flow past a shrinking/stretching rotating disk with variable thermal fluid properties. The MATLAB bvp4c function for numerical results is used. Characterization of viscous liquid flow due to the oscillation of disk with variable thermal conductivity is discussed by Rauf et al.^42^. Lately, researchers have shown interest in many aspects of variable thermal conductivity flow^43–48^.

The aforementioned studies disclosed that abundant researches are available in the literature discussing the nanofluid flows with variable thermal conductivity in numerous geometries. However, no work so far is conducted that deliberates the nanoliquid flow with temperature-dependent viscosity and variable thermal conductivity simultaneously in the presence of the C–C heat flux and gyrotactic motile organisms over a rotating disk. The uniqueness of the model is made unique by addition the impacts of the non-uniform source/sink and the chemical reaction. The problem is handled numerically. The graphs are plotted for numerous parameters versus involved profiles. Table 1 is displayed to distinguish the presented model from the published works. A clear picture is portrayed here for the uniqueness of the existing study.

**Table 1:** A comparative analysis of the presented model with the published works.

References	Variable viscosity	Variable Thermal conductivity	Non-uniform heat source/sink	Cattaneo Christov heat flux	Nanofluid flow	Entropy generation	Convective boundary conditions
Bhandari et al.^51^	Yes	Yes	No	No	Yes	Yes	No
Nadeem et al.^52^	No	No	No	No	Yes	Yes	No
Riasat et al.^53^	Yes	Yes	No	No	Yes	No	No
Wakeel et al.^54^	No	No	No	Yes	Yes	Yes	No
Rout et al.^55^	No	No	No	No	Yes	Yes	No
Present	Yes	Yes	Yes	Yes	Yes	Yes	Yes

## Mathematical modeling

**Figure 1:**
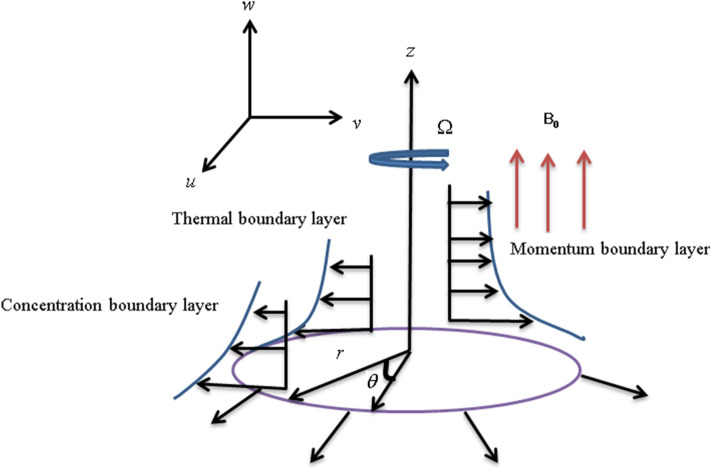
Sketch of the coordinates system.

Subsequent assumptions are considered for the proposed model as under:

1. The nanofluid flow is considered over a rotating disk.
2. A three-dimensional axisymmetric nanofluid flow with velocities $(u,v,w)$ is taken in $(r,\varphi ,z)$ directions.
3. The nanofluid flow is considered under a strong magnetic field.
4. The axis of the disk with angular velocity $r\Omega$ is considered in the z-direction.
5. The flow is under the convective mass and heat boundary conditions.
6. The consideration of the variable thermal conductivity with C–C heat flux and viscosity is also a part of the project model.
7. The proposed model is discussed for liquid and gas scenarios.
8. The influences of the non-uniform source/sink and the chemical reaction are also considered here.
9. The projected model is displayed in Fig. 1.

The boundary layer governing equations under the impacts of C–C heat flux, chemical reaction, and non-uniform heat generation/absorption is represented as^49,50^:

1 $$ \nabla .V = 0, $$

2 $$ \rho \left[ {({\mathbf{V}}.\nabla ){\mathbf{V}} + 2\Omega \times {\mathbf{V}} + \Omega \times \left( {\Omega \times {\mathbf{r}}} \right)} \right] = - \nabla p + \nabla .\tau - - \sigma B_{0}^{2} {\mathbf{V}}, $$

3 $$ \left( {{\mathbf{V}}.\nabla T} \right) + \frac{1}{{\left( {\rho c_{p} } \right)}}\nabla .{\mathbf{q}} = \nabla .\left( {K\left( T \right)\nabla T} \right) + \tau \left( {D_{B} \nabla \phi + \frac{{D_{T} }}{{T_{\infty } }}\nabla T} \right)\nabla T + q*. $$

The non-uniform heat source/sink is defined as:

4 $$ q* = \frac{{k_{\infty } U_{w} }}{{x\nu (\rho c_{p} )_{f} }}\left[ {A*(T_{w} - T_{\infty } )f + B*(T - T_{\infty } )} \right]. $$

The heat flux ${\mathbf{q}}$ satisfies the ensuing equation:

5 $$ {\mathbf{q}} + \lambda_{2} (\frac{{\partial {\mathbf{q}}}}{\partial t} - {\mathbf{q}}.\nabla {\mathbf{V}} + (\nabla .{\mathbf{V}}){\mathbf{q}} + {\mathbf{V}}.\nabla {\mathbf{q}}) = - K(T)\nabla .T. $$

The Eq. (3) may be transformed into classical Fourier's law when the relaxation time of heat flux $\lambda_{2} = 0$. After the Elimination of ${\mathbf{q}}$ from Eq’s $(3)$ and $(5)$, we obtain

6 $$ \begin{aligned} (\rho c_{p} )_{f} \left( {{\mathbf{V}}.\nabla T} \right) + \lambda_{2} \Omega_{2} & = \nabla .\left( {K\left( T \right)\nabla T} \right) + (\rho c_{p} )_{f} \left( {D_{B} \nabla C + \frac{{D_{T} }}{{T_{\infty } }}\nabla T} \right)\nabla T \hfill \\&\quad + \frac{{k_{\infty } U_{w} }}{{x\nu (\rho c_{p} )_{f} }}\left[ {A*(T_{w} - T_{\infty } )f + B*(T - T_{\infty } } \right], \hfill \\ \end{aligned} $$

where $\Omega_{2}$ is defined as:

7 $$ \Omega_{2} = u\frac{\partial w}{{\partial r}}\frac{\partial T}{{\partial z}} + w\frac{\partial w}{{\partial z}}\frac{\partial T}{{\partial z}} + u^{2} \frac{{\partial^{2} T}}{{\partial r^{2} }} + w^{2} \frac{{\partial^{2} T}}{{\partial z^{2} }} + 2wu\frac{{\partial^{2} T}}{\partial r\partial z} + u\frac{\partial u}{{\partial r}}\frac{\partial T}{{\partial r}} + w\frac{\partial u}{{\partial z}}\frac{\partial T}{{\partial r}}. $$

8 $$ {\varvec{V}}\nabla C = \nabla \left( {D_{B} \nabla C + \frac{{D_{T} }}{{T_{\infty } }}\nabla T} \right) - k_{r}^{2} \left( {C - C_{\infty } } \right). $$

The boundary conditions assisting the devised model are:

9 $$ u = 0,v = 0,w = 0, - K(T)T_{z} = h_{f} \left[ {T_{w} - T} \right],\,\,\,\, - D_{B} C_{z\,} = h_{s} \left[ {C_{w} - C} \right],\,{\text{at}}\quad \eta \to 0. $$

10 $$ u \to 0,v \to - 1,T \to 0,\,\,C \to 0,\;{\text{as}}\;\eta \to \infty . $$

In the above model, equations ${\varvec{r}}$ signify the position vector with components ${\varvec{r}} = (r,0,z)$ ,${\mathbf{V}}$ is the velocity vector of the fluid with components ${\mathbf{V = }}(u,v,w)$ and ${{\varvec{\Omega}}} = (0,0,r\Omega )$ is the angular velocity of the disk. The stress tensor with its components for the generalized fluid is given by:

$$ \tau_{rr} = 2\mu (T)\frac{\partial u}{{\partial r}}\;\tau_{\varphi \varphi } = \mu (T)\frac{u}{r},\;\tau_{\varphi \varphi } = \mu (T)\frac{u}{r},\;\tau_{zz} = 2\mu (T)\frac{\partial w}{{\partial z}}, $$

11 $$ \tau_{\varphi z} = \tau_{z\varphi } = \mu (T)\frac{\partial v}{{\partial z}},\;\tau_{rz} = \tau_{zr} = \mu (T)\left( {\frac{\partial u}{{\partial z}} + \frac{\partial w}{{\partial r}}} \right),\;\tau_{r\varphi } = \tau_{\varphi r} = \mu (T)\left( {r\frac{\partial }{\partial r}\left( \frac{v}{r} \right)} \right), $$

$\mu (T)$ illustrated the variable viscosity, which is dependent on temperature and is defined by^50^:

12 $$ \mu (T) = \frac{{\mu_{\infty } }}{{1 + \lambda \left( {T - T_{\infty } } \right)}}, $$

Here, $\mu_{\infty }$ represents the free stream viscosity. The value of $\lambda$ classifies the deliberation of viscosity for thermal differences. As for $\lambda < 0$ expressing gas type fluids while liquid characteristics for $\lambda > 0$. For Newtonian fluid $\lambda = 0$. Similarly, the variable thermal conductivity is defined by^50^:

13 $$ K(T) = \kappa_{\infty } \left( {1 + \varepsilon \frac{{T - T_{\infty } }}{{T_{w} - T_{\infty } }}} \right), $$

$\kappa_{\infty }$ is the free stream thermal conductivity. Fluids e.g., water and air thermal conductivity parameter is $\varepsilon > 0$. Nevertheless $\varepsilon < 0$ represents the fluids e.g., lubricating oil.

14 $$ \begin{gathered} f = \frac{u}{r\Omega },g = \frac{v}{r\Omega },H = \frac{w}{l\Omega },P = \frac{p}{{\rho \left( {\Omega l} \right)^{2} }},\eta = z\sqrt {\frac{\Omega }{\nu }} , \hfill \\ \theta = \frac{{T - T_{\infty } }}{{T_{w} - T_{\infty } }},\phi = \frac{{C - C_{\infty } }}{{C_{w} - C_{\infty } }},l = \sqrt {\frac{\nu }{\Omega }} \hfill \\ \end{gathered} $$

By invoking the above transformation into the Eqs. (2)–(9) we have the following equations.

15 $$ 2f + H^{\prime} = 0, $$

16 $$ f^{2} - (g + 1)^{2} + f^{\prime}H - \left( {\mu (T)f^{\prime}} \right)^{\prime } - \frac{Mf}{{1 + \lambda \theta }} = 0, $$

17 $$ 2f\left( {g + 1} \right) + g^{\prime}H - \left( {\mu (T)g^{\prime}} \right)^{\prime } - \frac{Mg}{{1 + \lambda \theta }} = 0, $$

18 $$ H\theta^{\prime} - \frac{1}{\Pr }\left( {\theta^{\prime}K(T)} \right)^{\prime } + N_{b} \theta^{\prime}\phi^{\prime} + N_{t} \theta^{2\prime } - \gamma_{1} \left( {H^{2} \theta^{\prime\prime} + HH^{\prime}\theta^{\prime}} \right) + \left( {1 + \varepsilon \theta } \right)\left( {Af + B\theta } \right) = 0, $$

19 $$ \phi^{\prime\prime} + Scf\phi^{\prime} + \frac{{N_{t} }}{{N_{b} }}\theta^{\prime\prime} - ScK_{c} \phi = 0, $$

The boundary conditions are obtained in the following form:

$$ \begin{gathered} H(0) = 0,\,\,\,g(0) = 0,\,\,\,\,f(0) = 0,\,\,\theta^{\prime}(0) = B_{2} \left( {\frac{1 - \theta (0)}{{1 + \varepsilon \theta }}} \right),\,\,\,\,\,\phi^{\prime}(0) = B_{1} (1 - \phi (0)) \hfill \\ \hfill \\ \end{gathered} $$

20 $$ f \to 0,g \to - 1,\theta \to 0,\,\,\phi \to 0, $$

Substituting Eqs. (12) and (13) in Eqs. (16), (17), and (18), we have the following form of the equations:

21 $$ 2f + H^{\prime} = 0, $$

22 $$ f^{\prime}H - (g + 1)^{2} + f^{2} - \left( { - \frac{{\lambda \theta^{\prime}}}{{(1 + \lambda \theta )^{2} }}f^{\prime} + \frac{{f^{\prime\prime}}}{(1 + \lambda \theta )}} \right) - Mf = 0, $$

23 $$ 2f\left( {g + 1} \right) + g^{\prime}H - \left( { - \frac{{\lambda \theta^{\prime}}}{{(1 + \lambda \theta )^{2} }}g^{\prime} + \frac{{g^{\prime\prime}}}{(1 + \lambda \theta )}} \right) - Mg = 0, $$

24 $$ H\theta ^{\prime }  - \frac{1}{{\Pr }}\left( {\varepsilon \theta ^{{\prime 2}}  + (1 + \varepsilon \theta )\theta ^{{\prime \prime }} } \right) + N_{b} \theta ^{\prime } \phi ^{\prime }  + N_{t} \theta ^{{2\prime }}  - \gamma \left( {H^{2} \theta ^{{\prime \prime }}  + HH^{\prime } \theta ^{\prime } } \right) + \left( {1 + \varepsilon \theta } \right)\left( {Af + B\theta } \right) = 0, $$

25 $$ \phi^{\prime\prime} - ScH\phi^{\prime} + \frac{{N_{t} }}{{N_{b} }}\theta^{\prime\prime} - ScK_{c} \phi = 0, $$

The boundary conditions are obtained in the following form:

$$ \begin{gathered} H(0) = 0,\,\,\,g(0) = 0,\,\,\,\,f(0) = 0,\,\,\theta^{\prime}(0) = B_{2} \left( {\frac{1 - \theta (0)}{{1 + \varepsilon \theta }}} \right),\,\,\,\,\,\phi^{\prime}(0) = B_{1} (1 - \phi (0)) \hfill \\ \hfill \\ \end{gathered} $$

26 $$ f \to 0,g \to - 1,\theta \to 0,\,\,\phi \to 0, $$

The above quantities are defined as:

27 $$ \begin{gathered} Sc = \frac{\nu }{{D_{B} }},\Pr = \frac{{c_{p} \mu_{\infty } }}{{\kappa_{\infty } }},\gamma = \lambda_{1} \Omega ,\,\,\,\,N_{b} = \frac{{\tau D_{B} (C_{w} - C_{\infty } )}}{\nu },\,\,N_{t} = \frac{{\tau D_{T} \Delta T}}{{T_{\infty } \nu }},\,\,\,\,\,\,\, \hfill \\ \,B_{2} = \frac{{h_{f} }}{{\kappa_{\infty } }}\sqrt {\frac{\nu }{\Omega }} ,\,\,\,K_{c} = \frac{{k_{r}^{2} }}{\Omega },\,\,\,B_{1} = \frac{{h_{s} }}{{\kappa_{\infty } }}\sqrt {\frac{\nu }{\Omega }} ,\, \hfill \\ \hfill \\ \end{gathered} $$

Mathematically the heat transfer rate is defined as:

28 $$ Nu_{r} = \frac{{hq_{w} }}{{k_{\infty } (T_{w} - T_{\infty } )}} $$

where

29 $$ q_{w} = - K(T)\left. {T_{z} } \right|_{z = 0} $$

Substituting Eq. (29) into Eq. (28), we have

30 $$ Nu_{r} = - (1 + \varepsilon \theta )\theta ^{\prime}(0) $$

## Entropy generation analysis

Following the volumetric entropy generation is given as:

31 $$ \begin{aligned} S_{G} & = \frac{K(T)}{{T_{\infty }^{2} }}\left( {T_{z} } \right)^{2} + \frac{\mu (T)}{{T_{\infty } }}\left( {2[u_{r}^{2} + \frac{1}{{r^{2} }}u^{2} + w_{z}^{2} ] + v_{z}^{2} + u_{z}^{2} + (r(\frac{v}{r})_{r} )^{2} } \right) \hfill \\&\quad + \frac{{\sigma B_{0}^{2} }}{{T_{\infty } }}\left( {u^{2} + v^{2} } \right) + \frac{{R_{D} }}{{T_{\infty } }}T_{z} C_{z} + \frac{{R_{D} }}{{C_{\infty } }}(C_{z} )^{2} . \hfill \\ \end{aligned} $$

The entropy generation in the dimensionless form can be derived as:

32 $$ \begin{aligned} N_{G} & = (1 + \varepsilon \theta )\alpha \theta ^{{\prime}{2}} + \frac{Br}{{1 + \lambda \theta }}\left( {\frac{12}{{\text{Re}}}\left\{ {f^{2} + 2R^{2} \left\{ {f^{{\prime}{2}} + g^{{\prime}{2}} } \right\}} \right\}} \right) \hfill \\&\quad + M^{2} \left( {f^{2} + g^{2} } \right) + {\text{Re}} \sum \left( {\phi ^{{\prime}{2}} + \frac{\phi ^{\prime}\theta ^{\prime}}{\alpha }} \right), \hfill \\ \end{aligned} $$

where

33 $${\text{Re}} = \frac{{\Omega l^{2} }}{\nu },Br = \frac{{\mu_{\infty } \Omega^{2} l^{2} }}{{k_{\infty } (T_{w} - T_{\infty } )}},\Sigma = \frac{{R_{D} (C_{w} - C_{\infty } )}}{{C_{\infty } k_{\infty } }},\alpha = \frac{\Delta T}{{T_{\infty } }},R = \frac{r}{l},S_{0}^{{\prime\prime}{\prime}} = \frac{{k_{\infty } (T_{w} - T_{\infty } )}}{{\nu T_{\infty } }}. $$

## Numerical procedure

To obtain the solution of coupled nonlinear by employing bvp4c with MATLAB, the first step is to obtain the first-order differential equations with newly defined variables:

34 $$ \begin{gathered} f = y_{1} ,f^{\prime} = y_{2} ,f^{\prime\prime} = yy_{1} ,g = y_{3} ,g^{\prime} = y_{4} ,g^{\prime\prime} = yy_{2} , \hfill \\ H^{\prime} = yy_{3} ,\theta = y_{6} ,\theta^{\prime} = y_{7} ,\theta^{\prime\prime} = yy_{4} \hfill \\ \phi = y_{8} ,\phi^{\prime} = y_{9} ,\phi^{\prime\prime} = yy_{4} . \hfill \\ \end{gathered} $$

Using Eq. (34), Eqs. (21)–(25) together with boundary conditions Eq. (26) take the following form:

35 $$ \begin{gathered} yy_{1} = \, \left[ {y_{5} y_{2} - y_{1}^{2} + \left( {1 + y_{3} } \right)^{2} + \frac{{\lambda y_{7} y_{2} }}{{\left( {1 + \lambda y_{6} } \right)^{2} }} - My_{1} } \right]\left( {1 + \lambda y_{6} } \right), \hfill \\ \, \hfill \\ \end{gathered} $$

36 $$ yy_{2} = \left[ {2y_{1} y_{3} + y_{5} y_{4} + 2y_{1} + \lambda \left( {1 + \lambda y_{6} } \right)^{ - 2} y_{7} y_{4} + M\,y_{4} } \right]\left( {1 + \lambda y_{6} } \right), $$

37 $$ yy_{3} = - 2y_{1} , $$

38 $$ yy_{4} \, = \frac{{\Pr y_{5} y_{7} - \varepsilon y_{7}^{2} + Ay_{1} + By_{5} + N_{b} y_{6} y_{8} + N_{t} y_{6}^{2} }}{{\left( {1 + \varepsilon y_{6} + \Pr \gamma y_{5} y_{5} } \right)}}, $$

39 $$ yy_{5} = \left( { - Scy_{1} y_{8} - \frac{{N_{b} }}{{N_{t} }}yy_{4} + K_{c} y_{7} } \right), $$

40 $$ \begin{gathered} y_{1} (0) = 0,y_{3} (0) = 0,y_{5} (0) = 0,y_{6} (0) = B_{2} \left[ {\frac{{\left[ {1 - y_{5} (0)} \right]}}{{\left[ {1 + \varepsilon_{1} y_{5} \left( 0 \right)} \right]}}} \right], \hfill \\ y_{8} (0) = B_{1} \left[ {1 - y_{7} (0)} \right],y_{1} (\infty ) = 0,y_{3} (\infty ) = 1,y_{6} (\infty ) = 0,y_{8} (\infty ) = 1. \hfill \\ \end{gathered} $$

## Flow diagram of Numerical procedure

**Figure 2:**
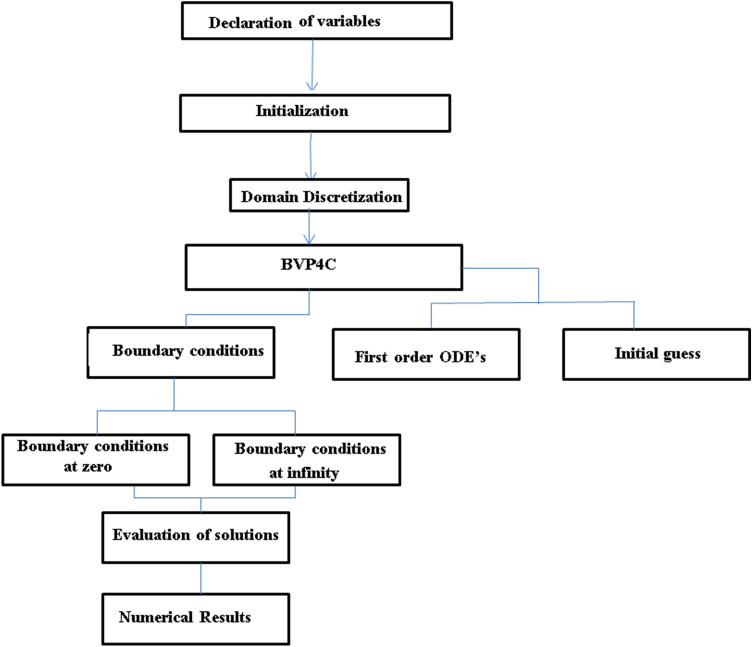
Flow chart of numerical procedure.

The whole procedure of the numerical method used is depicted in Fig. 2.

## Outcomes and discussion

**Figure 3:**
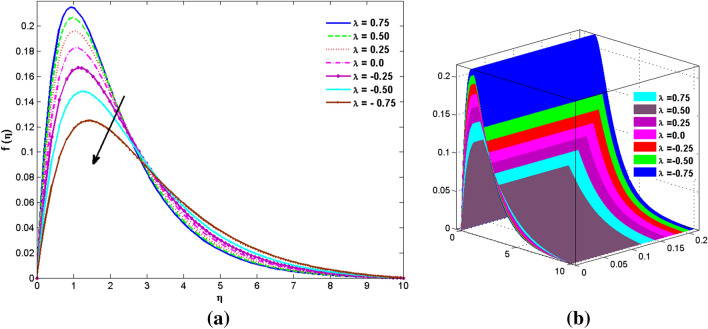
Variations of $\lambda$ to $f(\eta )$.

**Figure 4:**
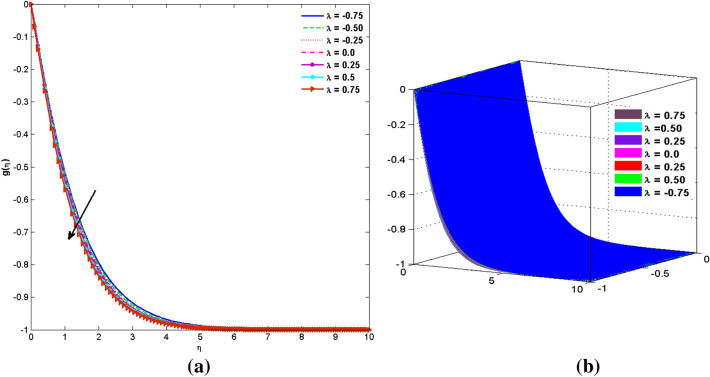
Variations of $\lambda$ to $g(\eta )$_._

**Figure 5:**
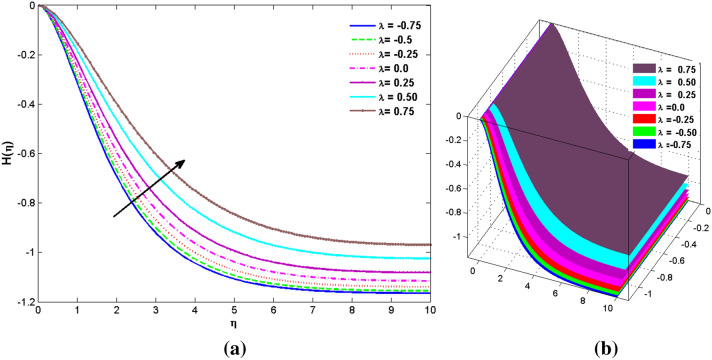
Variations of $\lambda$ to $H(\eta )$.

**Figure 6:**
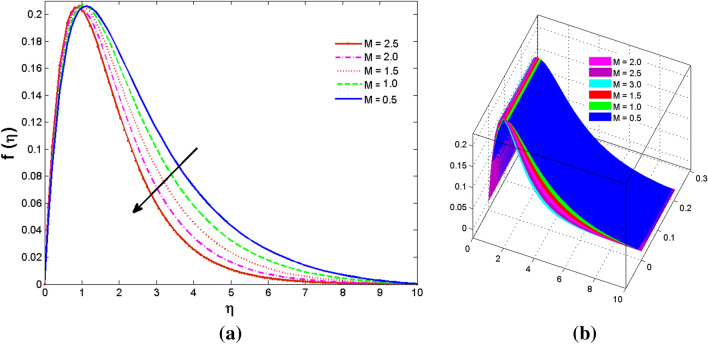
Variations of $M$ to $f(\eta )$_._

**Figure 7:**
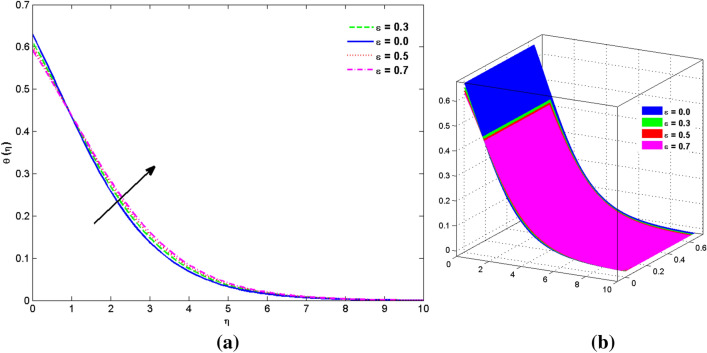
Variations of $\varepsilon$ to $\theta (\eta )$_._

**Figure 8:**
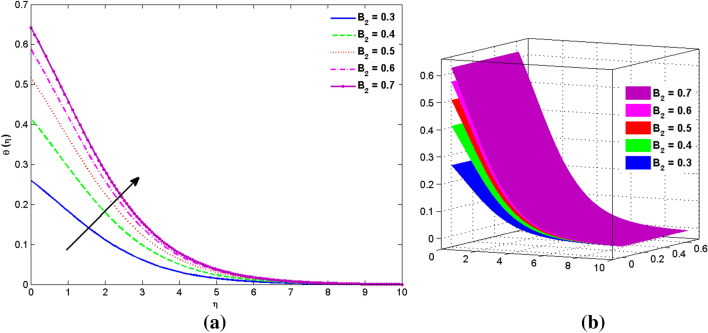
Variations of $B_{2}$ to $\theta (\eta )$_._

**Figure 9:**
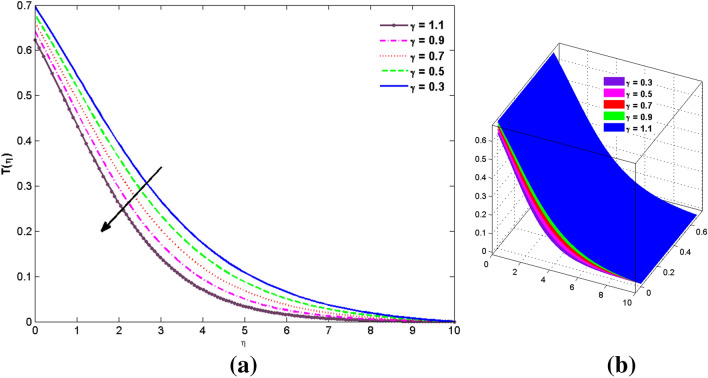
Variations of $\gamma$ to $\theta (\eta )$.

**Figure 10:**
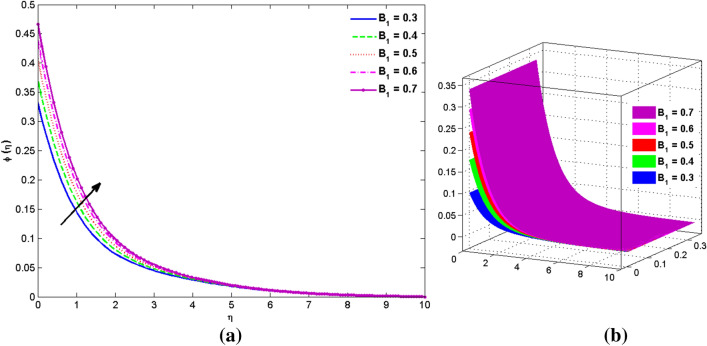
Variations of $B_{1}$ to $\phi (\eta )$_._

**Figure 11:**
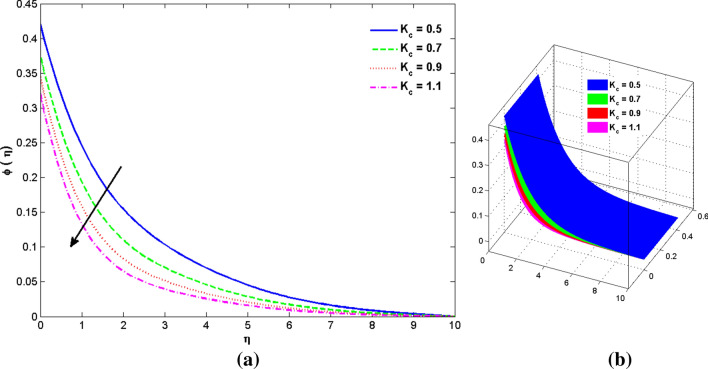
Variations of $K_{c}$ to $\phi (\eta )$_._

**Figure 12:**
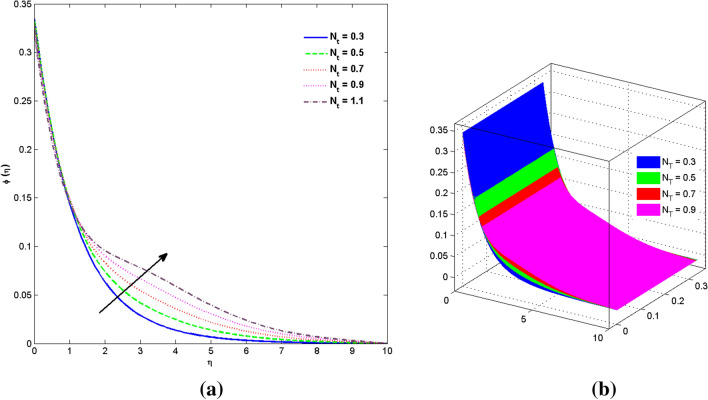
Variations of $N_{T}$ to $\phi (\eta )$_._

**Figure 13:**
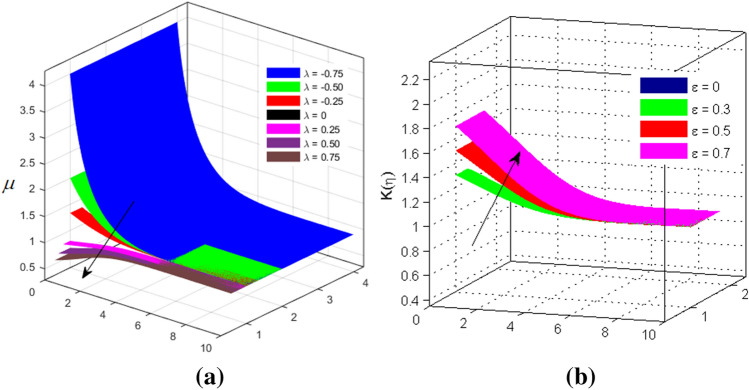
Variations of $\lambda$ and $\varepsilon$ to $\mu$ and $K(\eta )$ respectively.

**Figure 14:**
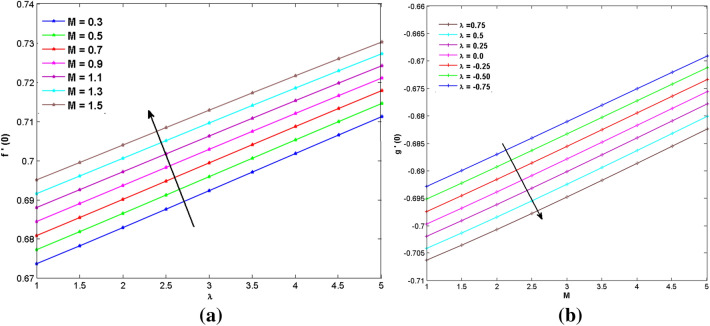
Profile of $f^{\prime}(0)$ and $g^{\prime}(0)$ versus $M$ for increasing values of $\lambda$.

**Figure 15:**
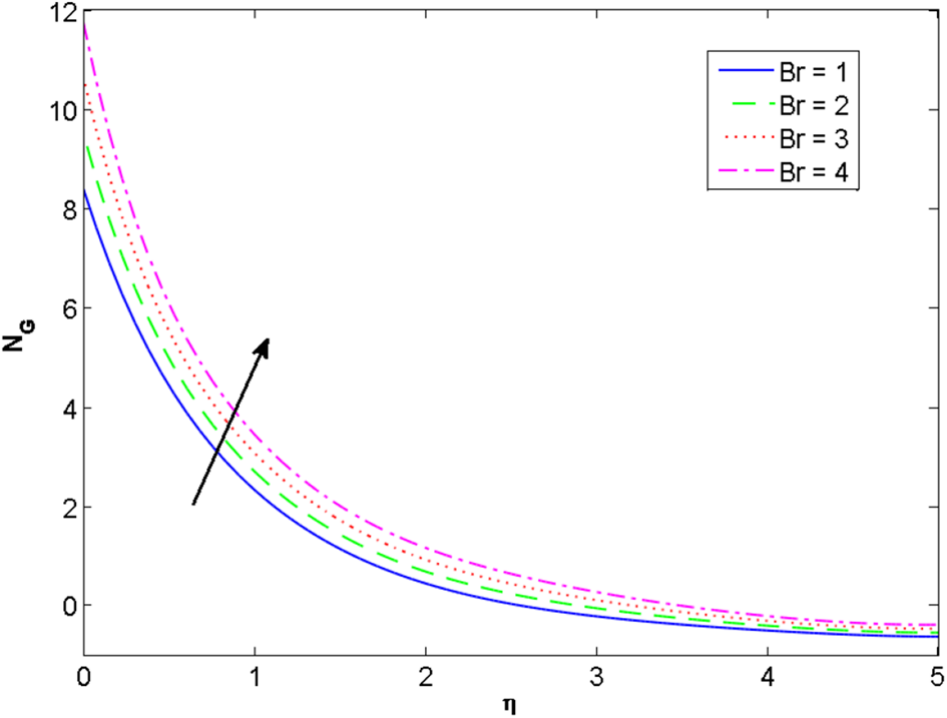
Profile of $N_{G}$ versus $Br$.

**Figure 16:**
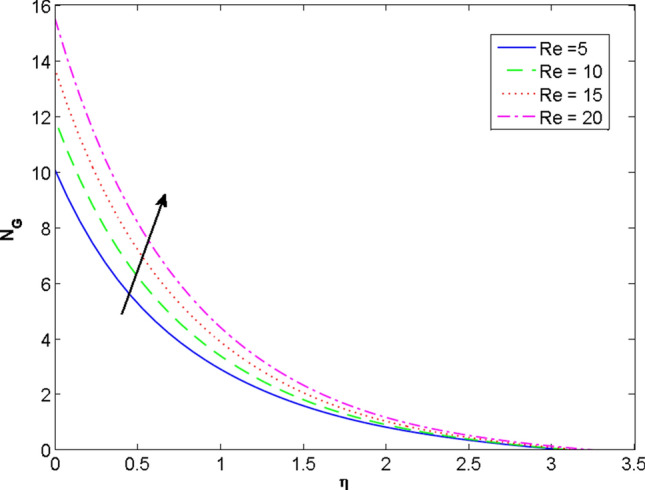
Profile of $N_{G}$ versus ${\text{Re}}$.

The two and three-dimensional view of the effect of the temperature-dependent constant of variable viscosity on the radial, azimuthal, and axial flow profiles is exhibited in Figs. 3a, 4, 5b. Figure 3a represents the radial flow profile for the parameter $- 0.75 \le \lambda \le 0.75$. A decline in amplitude owes to the fact of decrement in the boundary layer and the profile gets closer to the disk. Physically increasing values of $\lambda > 0$ (for liquids) for an isothermally heated disk causing the fluid to become less viscous near the disk. For $\lambda < 0$ (for gas) fluid becomes more viscous for incremental values of $\lambda$. A decrease in viscous interaction of fluid and disk surface and shear stress causes the decrease in the vicinity of the disk to move the fluid in the radial direction which consequently leads to the decline in radial profile. Figure 3b is the three-dimensional depiction of the same results. Figure 4a is drawn to visualize the mean flow phenomenon in the azimuthal direction for the variations of the parameter in the range $- 0.75 \le \lambda \le 0.75$. The distribution curves represent that convergence is attained at small values of $\eta$. The azimuthal velocity profile declines for higher estimation of $\lambda$. The amplitude declines sharply near the disk. Figure 3b is the three-dimensional flow behavior of Fig. 4a. Figure 5a demonstrates the axial flow for escalating values of $\lambda$. The fluid flow is occurring in the *z*-direction with angular velocity $\Omega$. The Coriolis and centrifugal forces arising due to the rotation. For $\lambda > 0$ (for liquids) increasing $\lambda$ fluid becomes less viscous which inhibits these Coriolis and centrifugal forces and radially outward flow of the fluid caused by these forces is reduced. For $\lambda < 0$ (for gas) fluid becomes more viscous for incremental values of $\lambda$. Thereby near the disk, the ability to push the fluid gets diminished and flow caused by centrifugal forces declines, and far away from the disk, these centrifugal forces cause the outward radial flow which is compensated by the inward axial flow and causes the escalation of axial flow. Figure 5b is the three-dimensional axial profile for the same parameters. The behavior of M on the velocity profile $f(0)$ is revealed in Fig. 6a,b. It is witnessed that, the velocity component is on the decline due to the strong Lorentz force which resists the fluid motion, and movement of fluid particles slow down that’s why velocity profile decrease. Figure 7a,b are sketches of thermal profiles for increasing thermal conductivity parameters. It is observed that the thermal profile increases for greater thermal conductivity. Because more amount of heat is being transferred from the disk surface to the fluid. Consequently, the escalating trend is seen. As we move far away from the disk the thermal conductivity reduces to constant thermal conductivity and hence stops the more increase in the thermal profile. The impact of the conjugate parameter $B_{2}$ on the thermal profile is studied in Fig. 8a,b. Since B is involved with heat transfer rate at the surface. Here, the enlargement in $B_{2}$ initiates the heat transfer that pushes extra heat from the surface. The thermal boundary layer improves as B increases, resulting in a temperature rise. Impact thermal relaxation parameter $\gamma$ on temperature field is shown in Fig. 9a,b. The thickness of the thermal boundary layer is likewise decreasing. In fact, higher values of $\gamma$ suggest that measured material particles need more opportunities to transmit heat to their neighboring particles. As a result, a higher estimates of are active in decreasing the thermal profile. For $\lambda = 0$ the heat is transferred immediately throughout the material. Therefore, the temperature profile is greater for $\lambda = 0$, i.e., for Fourier’s law of heat conduction when compared with C–C heat flux model. The influence of the convection–diffusion parameter $B_{1}$ on concentration distribution is shown in Fig. 10a,b. An increase in the concentration profile is attained with increasing $B_{1}$. The influence of the chemical reaction parameter $K_{c}$ against the concentration profile is depicted in Fig. 11a,b. It is noticed that the concentration of the fluid weakens once the estimates of $K_{c}$ are augmented. Large values of $K_{c}$ are associated with the destructive chemical reaction and this eventually dissolves the fluid species. That is why the concentration of the fluid is reduced. The effects of $N_{t}$ on concentration profile are depicted in Fig. 12a,b. The higher concentration is observed for higher $N_{t}$. Because greater $N_{t}$ push the nanoparticles away from the warm surface which causes enhancement in concentration profiles. Figure 13a represents the viscosity profile for increasing values of $\lambda$. The viscosity is maximum at the lowest value of $\lambda < 0$ (for gas). As we move far away from the vicinity of the disk the viscosity converges to the value of constant viscosity i.e., $\mu = 1$. For $\lambda > 0$ (for liquids) the viscosity curve increases and converges to the constant value $\mu = 1$. Near the disk the values of viscosity are less or greater than 1 depending upon the value of $\lambda$. Figure 13b is the thermal conductivity profile for the variation in the parameter of thermal conductivity. Increasing thermal conductivity is obvious for isothermally heated disk. As the value of $\varepsilon$ increases, thermal conductivity enlarges which ensures more conduction of heat from disk surface to the fluid. As we move away from the disk heat conduction phenomenon gradually converges to 1 Fig. 14a and Fig. 14b shows $f^{\prime}(0)$ and $g^{\prime}(0)$ versus magnetic parameter with increasing values of $\lambda$ in the range $- 0.75 \le \lambda \le 0.75$. The increase in $f^{\prime}(0)$ and decline in $g^{\prime}(0)$ is witnessed. Figure 15, 16 display the vital role of volumetric entropy generation discussed for $Br$ and ${\text{Re}}$. For greater $Br$ and ${\text{Re}}$ entropy generation is increases. In detail for greater values of (Br) viscous dissipation generate less transfer rate and thus augments entropy generation rises. For $Br = 0,$ viscous dissipation, irreversibility disappears and only heat transfer irreversibility produce. The effect of *Re* on entropy generation is expressed in Fig. 16. For large estimates of the Reynolds number, the substantial motion of the fluid molecules is witnessed. Thus, escalating entropy generation rate. Table 2 depicts the comparison values of and for numerous estimates of the with Miller et al.^49^ and Mair et al.^50^. An excellent association is revealed. The Numerical outcomes of the Nusselt number for different value of $\varepsilon ,\gamma ,N_{T}$ and $B_{2}$ are presented in Table 3. It is observed that the heat transfer rate is decreases for greater values of $\varepsilon ,\gamma ,N_{T}$ while increases for $B_{2}$. The Grid free analysis Nusselt number are given in Table 4.

**Table 2:** Comparison of $g^{\prime}(0)$ and $f^{\prime}(0)$ with previous results of Miller et al.^49^ and Mair et al.^50^ when $\gamma = 0.0,B_{1} = 0.0,B_{2} = 0.0,K_{c} = 0.0,,\Pr = 0.72,M = 0.0.$

$\lambda$	^49^	^50^	Presents results	^49^	^50^	Presents results
	$f^{\prime}(0)$	$f^{\prime}(0)$	$f^{\prime}(0)$	$g^{\prime}(0)$	$g^{\prime}(0)$	$g^{\prime}(0)$
** − **0.75	0.2282	0.2281	0.2280	− 0.2216	− 0.2216	− 0.2217
** − **0.50	0.3520	0.3520	0.3519	− 0.3896	− 0.3896	− 0.3898
** − **0.25	0.4397	0.4397	0.4396	− 0.5136	− 0.5136	− 0.5138
0.00	0.5102	0.5102	0.5101	− 0.6159	− 0.6159	− 0.6161
0.25	0.5710	0.5707	0.5705	− 0.7022	− 0.7036	− 0.7038
0.50	0.6254	0.6254	0.6253	− 0.7773	− 0.7774	− 0.7776
0.75	0.6753	0.6752	0.6751	− 0.8844	− 0.8845	− 0.8847

**Table 3:** Numerical outcomes of the heat transfer rate $(Re_{x} )^{ - 0.5} Nu_{x}$ for different value of $\varepsilon ,\gamma ,N_{T}$ and $B_{2}$.

$\varepsilon$	$\gamma$	$N_{T}$	$B_{2}$	$(Re_{x} )^{ - 0.5} Nu_{x}$
0.1	0.5	0.7	0.5	0.1285
0.3				0.1203
0.5				0.1136
0.5	0.1			0.1341
	0.3			0.1274
	0.5			0.1212
		0.3		0.1223
		0.5		0.1179
		0.7		0.1136
			0.6	0.1173
			0.7	0.1201
			0.8	0.1223

**Table 4:** Grid free analysis for heat transfer rate.

Serial no.	Grid size	$Nu_{ave,\theta }$
1	10 × 10	0.3470
2	20 × 20	0.2706
3	50 × 50	0.2251
4	100 × 100	0.1765
5	200 × 200	0.1744
6	300 × 300	0.1741
7	500 × 500	0.1741
8	100 × 100	0.1741

## Concluding comments

The present investigation has elucidated to witness the impact of variable thermal conductivity and variable viscosity on the flow field generated by the rotating disk with MHD, chemical reaction with C–C heat flux. The convective mass and heat boundary conditions are assumed to support the envisioned problem. The problem is solved numerically. Three main findings have emerged as follows:

- In a gas-type fluid, the boundary layer flow is concentrated near the disk and it is improved with enhancement in the temperature.
- The radial velocity declines positive estimates of the viscosity for thermal variations and an opposite trend is witnessed for the negative values.
- Thermal boundary layer thickness is maximum in case of higher thermal conductivity.
- Both thermal and concentration profiles escalate for large estimates of the respective conjugate parameters.
- Higher estimations of the $\gamma$ cause reduction in the thermal profile.
- The fluid radial velocity is increased for mounting estimates of wall magnetic parameters.

## List of symbols

*Sc*: Schmidt number
*c*_*p*_: Capacity of specific heat $({\text{Jkg}}^{ - 1} \,{\text{K}}^{ - 1} )$
$C_{\infty }$: Concentration in the free stream $({\text{mol}}\,{\text{m}}^{ - 3} )$
*B*_*2*_: Convection thermal parameter
(*u*, *v*, *w*): Velocity components $$({\text{m}}\,{\text{s}}^{ - 1} )$$
$\kappa_{\infty }$: Free stream thermal conductivity $({\text{W}}\,{\text{m}}^{ - 1} \,{\text{K}}^{ - 1} )$
$q*$: Non-uniform heat source/sink
r: Radius $$({\text{m}})$$
$T_{w}$: Temperature on wall
$\gamma_{1}$: Thermal relaxation time $({\text{m}}\,{\text{s}}^{ - 1} )$
$$\Omega$$: Rotation parameter $$({\text{T}}^{ - 1} )$$
$\rho$: Density of fluid $({\text{kg}}/{\text{m}}^{3} )$
*D*_*T*_: Coefficient of thermophoretic diffusion $({\text{m}}^{2} \,{\text{s}}^{ - 1} )$
Pr: Prandtl number
**V**: Velocity vector of the fluid $({\text{m s}}^{ - 1} )$
*B*_*1*_: Convection diffusion parameter
*R*: Dimensionless radius $$({\text{m}})$$
Re: Reynolds number
*σ*: Electrical conductivity $$({\text{sm}}^{ - 1} )$$
*C*_*w*_: Wall concentration $$({\text{mol m}}^{ - 3} )$$
*k*_*r*_^*2*^: Reaction rate $$({\text{T}}^{ - 1} )$$
*A*,*B*: Heat generation and absorption parameters of Space-dependent and temperature-dependent
*h*_*s*_: Mass transfer coefficient $({\text{Wm}}^{ - 2} \,{\text{K}}^{ - 1} )$
_*C*_: Fluid concentration $$({\text{mol m}}^{ - 3} )$$
*T*: Fluid temperature $$({\text{K}})$$
*N*_*b*_: Brownian motion parameter
$\mu_{\infty }$: Free stream viscosity $({\text{ms}}^{ - 1} )$
$A*,B*$: Source, sink coefficients
*λ*: Viscosity thermal variation $({\text{m s}}^{ - 1} )$
*N*_*t*_: Thermophoresis parameter
*μ*: Liquid dynamic viscosity $({\text{m}}^{2} {\text{ s}}^{ - 1} )$
*λ*_2_: Thermal relaxation factor $({\text{m s}}^{ - 1} )$
$$\nu$$: Kinematic viscosity $$({\text{m}}^{{2}} {\text{ s}}^{ - 1} )$$
*D*_*B*_: Coefficient of Brownian motion $({\text{m}}^{{2}} {\text{ s}}^{ - 1} )$
**q**: Heat flux $$({\text{W m}}^{ - 2} )$$
*ε*: Thermal conductivity parameter $({\text{W m}}^{ - 1} \,{\text{K}}^{ - 1} )$
*α*: Dimensionless temperature difference $({\text{m}})$
Σ: Diffusion constant
Br: Brinkman number
P: Pressure (Pa)
$$T_{\infty }$$: Ambient temperature $$({\text{K}})$$
